# Integrative Multi-Omics Reveal the Molecular Architecture and Systemic Burden of Metabolic Syndrome in a Brazilian Cohort

**DOI:** 10.3390/biology15201838

**Published:** 2026-10-14

**Authors:** Carlos Vinicius Ferreira da Silva, Carlos José Ferreira da Silva, Thais Regiani Cataldi, Carlos Labate, Youssef Bacila Sade, Sandra Mara Naressi Scapin, Fabiano Lopes Thompson, Cristiane Carneiro Thompson, Eidy de Oliveira Santos

**Affiliations:** 1 Institute of Biology, Federal University of Rio de Janeiro (UFRJ), Rio de Janeiro 21941-909, RJ, Brazil; fabianothompson1@gmail.com (F.L.T.); thompson.cristiane@gmail.com (C.C.T.); 2 Faculty of Biological Sciences and Health, Rio de Janeiro State University (UERJ), Rio de Janeiro 20550-013, RJ, Brazil; 3 Department of Genetics, Afya-University of Grande Rio (Afya-Unigranrio), Duque de Caxias 25075-142, RJ, Brazil; carlosjosebiology@gmail.com; 4 Department of Plant Genetics, Luiz de Queiroz College of Agriculture, University of São Paulo—USP, Piracicaba 09913-030, SP, Brazil; thais.cataldi@usp.br (T.R.C.); calabate@usp.br (C.L.); 5 Laboratory of Macromolecules and Biochemistry, National Institute of Metrology, Quality and Technology (INMETRO), Duque de Caxias 20261-232, RJ, Brazil; yousade@gmail.com (Y.B.S.); sandra.scapin@gmail.com (S.M.N.S.)

**Keywords:** metabolic disease, obesity, multi-omics, metabolism, proteomics, metabolomics, lipidomics

## Abstract

Metabolic Syndrome (MetS) includes a complex cluster of metabolic disorders that significantly increases the risk for cardiovascular disease and Type 2 Diabetes Mellitus. This study aimed to elucidate the molecular architecture and systemic burden of MetS by integrating high-resolution plasma proteomics, metabolomics, and lipidomics. A cohort of 60 adult volunteers (n = 37 controls; n = 23 MetS) from Brazil was subjected to comprehensive clinical evaluation and multi-omics profiling. Clinical analysis identified HOMA-IR and triglycerides as primary predictors of MetS. FDR was used for multiple testing correction for all data. Untargeted proteomics identified 589 proteins, with 26 showing significant differential abundance, including the upregulated Intercellular Adhesion Molecule-1 (ICAM-1) and Vanin-1 and the depleted protective Apolipoprotein D (ApoD) in MetS. Metabolomic analysis resulted in the identification of 410 metabolites, with 27 distinct features, revealing accumulation of branched-chain and aromatic amino acids, alongside a significant reduction in the signaling molecule 3-hydroxybutyrate, in the case group. Lipidomic profiling identified 1241 lipids, with 41 differentially abundant, highlighting the increase in Phosphatidylinositol (PI 40:5) and Phosphatidylcholine (PC) classes and the depletion of Sphingomyelins (SM). Integrative analysis demonstrated that MetS comprises a synchronized and multisystemic molecular disruption rather than a collection of independent risk factors. The homeostatic breakdown is characterized by the depletion of the body’s antioxidant and anti-inflammatory defense, and the elevation of pro-inflammatory mediators like ICAM-1 and Vanin-1, the accumulation of lipotoxic drivers and a significant disruption in amino acid catabolism (BCAA) that overactivates the mTORC1 pathway. These findings provide a multifactorial metabolic dysregulation, offering putative signatures for future diagnoses and risk stratification.

## Simple Summary

Metabolic Syndrome (MetS) is a complex cluster of metabolic disorders that increases the risk of cardiovascular disease and type 2 diabetes. To elucidate its molecular architecture, a study involving 60 adults in Brazil (37 controls and 23 with MetS) integrated clinical, proteomic, metabolomic, and lipidomic analyses. Clinically, HOMA-IR and triglycerides emerged as primary predictors. Proteomic profiling revealed elevated pro-inflammatory markers (ICAM-1 and Vanin-1) and depleted protective ApoD. Metabolomics indicated accumulation of branched-chain and aromatic amino acids, along with decreased 3-hydroxybutyrate. Lipidomics showed an increase in phosphatidylinositols and phosphatidylcholines and a reduction in sphingomyelins. Integrative analysis demonstrated that MetS comprises synchronized and multisystemic molecular dysfunction, characterized by reduction in antioxidant defenses, elevation of inflammatory mediators, lipotoxicity and altered amino acid metabolism consistent with mTORC1 pathway activation. These findings provide a multifactorial molecular signature for diagnosis improvement and the development of personalized therapies.

## 1. Introduction

The rise of chronic metabolic diseases represents one of the greatest public health challenges of the 21st century, with MetS at the epicenter of this scenario. Defined as a cluster of disorders including visceral obesity, arterial hypertension, hyperglycemia, and dyslipidemia, MetS acts as a strong predisposing factor for the development of cardiovascular diseases and Type 2 Diabetes Mellitus (T2DM) [1,2]. While MetS is characterized by a state of insulin resistance and low-grade systemic inflammation, T2DM manifests when functional failure of pancreatic β-cells prevents compensation for this resistance, resulting in chronic hyperglycemia and multi-organ damage [3]. Instead, Reaven (1988) [1] proposed that MetS and T2DM are not separate entities, but continuous stages of the same pathophysiological spectrum driven by insulin resistance.

Epidemiologically, the global prevalence of MetS has doubled over the last two decades, affecting approximately 1.54 billion adults in 2023 [4]. On the American continent, and particularly in developing countries such as Brazil, the prevalence of metabolic syndrome is substantial, reaching 33% of the adult population, with 26% in men and 38% in women, according to a recent meta-analysis [5].

The economic burden imposed by these conditions is severe and threatens the sustainability of healthcare systems. Globally, diabetes-related expenditures alone exceeded US$1 trillion in 2024, representing approximately 12% of total global health spending [6]. Brazil ranks third worldwide in absolute costs of diabetes treatment, with an investment of US$45.1 billion in metabolic care in the past year [6]. Within the Brazilian Unified Health System (SUS), the presence of MetS increases treatment costs exponentially; individuals presenting all five components of the syndrome generate healthcare expenses approximately 700% higher than metabolically healthy individuals, largely driven by polypharmacy and specialized medical consultations [7]. Given this impact, a deep understanding of the molecular transition between the healthy state and metabolic dysregulation becomes imperative to enable early diagnosis.

Current methodologies, as high-resolution and multi-omics technologies, enable unprecedented investigation of the pathophysiology of MetS. Recent proteomics meta-analyses have identified a panel of 85 proteins consistently dysregulated in the plasma of patients with T2DM, involved in inflammatory pathways, lipoprotein organization, and immune response. Proteins such as LMAN2, APOA2, PSPA, and DLD, are associated with lipid and glucose metabolism [3]. Ferreira da Silva et al., 2024 and Da Silva et al. [8,9], 2025 observed a marked reduction in the protein ABCD4 (a lysosomal cobalamin transporter) and DMBT1 relative abundance, in individuals with MetS. Studies using high-sensitivity platforms have demonstrated that integrating 33 proteins into conventional risk models can increase predictive accuracy from 0.77 to 0.88 for T2DM development [10].

Metabolomics and lipidomics complement this molecular landscape by revealing the flux of small molecules and dysregulated lipid species. Elevated levels of branched-chain amino acids (BCAAs: leucine, isoleucine, and valine) are classical markers of insulin resistance, acting through excessive activation of the mTORC1 pathway [11]. Decreased levels of 3-hydroxybutyrate and 5-oxoproline were observed in individuals with MetS [12]. In the lipidomic context, MetS is characterized by a pro-inflammatory state, with the accumulation of phosphatidylinositol being associated with insulin resistance and obesity [13].

The transition toward precision medicine requires multi-omics integration to stratify patients into specific biological subgroups, enabling personalized interventions [14]. The early detection of molecular signatures provides a window of opportunity to mitigate vascular complications and reduce the human and financial burden imposed by MetS and T2DM [14,15].

In previous studies, we evaluated proteo-metabolomic profiles of individuals stratified by body mass index and metabolic health status (normal weight, overweight, obesity, and metabolic syndrome—MetS), in a Brazilian population [12]. In the present work, we expanded this investigation by adding lipidomics evaluation of plasma samples, by improving the MetS case group composition and focusing on a direct comparison against healthy controls. The case group here includes volunteers with MetS according to clinical criteria (National Cholesterol Education Program Adult Treatment Panel III) (NCEP/ATPIII), with and without Type 2 Diabetes Mellitus (T2DM) [15]. This cross-sectional study aims to deepen the understanding of MetS pathophysiology in a Brazilian population by integrating multi-omics approaches (proteomics, metabolomics, and lipidomics) in plasma samples. The study directly compares healthy control individuals with MetS volunteers to elucidate the molecular mechanisms and biological pathways involved in the progression of metabolic dysregulation.

## 2. Materials and Methods

### 2.1. Experimental Design

This study recruited 60 adult volunteers (≥18 years old) residing in Rio de Janeiro State, Brazil. This research was approved by the Ethics Committee in Human Research at Unigranrio, RJ, Brazil (approval number: 3402791) and was conducted following the ethical principles outlined in the Declaration of Helsinki (1964) and its subsequent amendments. All participants provided written informed consent prior to enrollment. Following a 12 h overnight fast, morning blood collection was performed in a clinical laboratory at the Afya-Unigranrio University, Duque de Caxias, RJ [12]. Participants were categorized into control group, without MetS (n = 37); and case group with MetS (n = 23). MetS was determined according to the NCEP/ATPIII protocol [15]. Volunteers were recruited based on clinical diagnostic criteria for Metabolic Syndrome, regardless of ongoing pharmacological regimens. Detailed records of baseline medication use were not systematically collected during clinical evaluation. To be classified with MetS, volunteers should have met at least 3 criteria established by the protocol [12]. Homoeostasis model assessment of insulin resistance (HOMA-IR) and β-cell function (HOMA-β) were conducted to supplement the clinical data associated with MetS [16,17,18]. All volunteers assigned to the MetS group (n = 23) strictly fulfilled the clinical diagnostic criteria according to the NCEP/ATPIII guidelines (≥3 risk factors). Of the 23 volunteers with MetS, 11 had pre-established diagnosis of T2DM, however independently met the NCEP/ATPIII criteria for MetS, ensuring that the entire case cohort shared a unified clinical and metabolic phenotype.

### 2.2. Clinical Evaluation

Clinical examinations were performed following established protocols [9,18]. The survey included BMI, height, weight, waist circumference, hip circumference, and waist-hip ratio, blood pressure evaluation using the oscillometric method (OMRON 7320) and biochemical blood analysis. Measurements with discrepancies exceeding 10 mmHg for systolic pressure or 5 mmHg for diastolic pressure were discarded to minimize errors [19]. Additionally, HOMA-IR and HOMA-β were calculated using previously described formulae [16,17].

### 2.3. Sample Processing

Plasma samples were obtained following established protocols [10]. Briefly, 5 mL of whole blood was collected intravenously after a 12 h fast in EDTA-containing tubes (Vacuplast EDTA K3) to prevent coagulation and stored at −80 °C. For protein purification, 50 μL aliquots of plasma were depleted of albumin and immunoglobulins using ProteoPrep Blue Albumin & IgG Depletion columns (Sigma-Aldrich, St. Louis, MO, USA) and eluted proteins were concentrated with Amicon Ultra-0.5 mL 3KD filter devices (Millipore Corporation, Billerica, MA, USA). Total protein concentration was determined by using the Bradford method.

### 2.4. Protein Processing for Mass Spectrometry

For mass spectrometry, 10 μg of proteins was subjected to an in-solution digestion protocol [20]. Protein complexes were denatured with 0.2% RapiGest (Waters, Milford, MA, USA) at 80 °C for 15 min, followed by reduction with 100 mM DTT at 60 °C for 30 min and alkylation with 300 mM iodoacetamide at room temperature for 30 min. Trypsin digestion was then performed overnight at 37 °C with a 1:100 (*w*/*w*) enzyme-to-protein ratio using sequencing-grade modified trypsin (Promega, Madison, WI, USA). The reaction was quenched with 5% (*v*/*v*) trifluoroacetic acid (TFA) at 37 °C for 90 min to hydrolyze RapiGest. Finally, the peptides were desalted using ZipTip C18 columns (Millipore Corporation) and resuspended in 0.1% TFA solution in (50%) acetonitrile (ACN) 70 μL that was lyophilized.

### 2.5. Protein Analysis by MS and Protein Identification

Mass spectrometry (MS) analysis was performed using a nanoElute nanoflow chromatography system from Bruker Daltonics (Bremen, Germany) coupled online to a hybrid trapped ion mobility spectrometry-quadrupole time-of-flight mass spectrometer (timsTOF Pro) from Bruker Daltonics. Mass spectrometry analysis of the samples and protein identification were performed according to previously described protocols [10]. Pooled quality control (QC) samples, prepared by combining equal aliquots from samples across groups, were also injected periodically between individual samples to monitor retention time stability and instrument performance.

MS raw data were processed using MaxQuant software version 2.4.0.0, specifically designed for mass spectrometry-based protein analysis [21]. The Andromeda search engine integrated into MaxQuant was employed [22]. The default configuration for data acquired on the TimsTOF Pro mass spectrometers was utilized. The fragment ion mass tolerance was set to 0.5 Da. Enzyme specificity was trypsin with a tolerance for peptides with up to two undigested cleavage sites. Methionine oxidation (15.994915 Da) and N-terminal protein acetylation (42.010565 Da) were defined as variable modifications, while cysteine carbamidomethylation (57.021464 Da) was defined as a fixed modification. The minimum peptide length was 7 amino acids. The peptide and protein false discovery rates (FDRs) were set to 1%. Additionally, at least one unique peptide was required for protein identification. Following this data processing step, relative abundance values for all identified proteins were obtained. The database used was the UniProt-reviewed Homo sapiens database, downloaded on 9 November 2023 with a total of 20,436 available proteins (https://www.uniprot.org/uniprotkb?query=humanþANDþ%28taxonomy_id%3A9606%29&facets=reviewed%3Atrue).

Perseus software version 2.0.9.0 [23] was employed for filtering the proteomics results. Proteins identified by only a modification site, as well as those identified by the reverse database and potential contaminants, were excluded from further analyses. Subsequently, an R programming (version 4.6.1) language script (https://www.R-project.org/) was used to refine the filter based on the percentage of protein presence in the groups and normalize the data by the total ion count (TIC).

### 2.6. Extraction of Plasma Metabolites

Metabolites were extracted using a 1:2 sample:extract solution ratio: 400 μL of ice-cold extraction mixture (acetonitrile:methanol, 1:1, *v*:*v*) in 200 μL sample. After agitation for 5 min, the samples were centrifuged (Eppendorf, Hamburg, Germany) at 15,000× *g* for 10 min at 4 °C for deproteinization. The supernatant fractions were then collected and evaporated to dryness using a vacuum concentrator (Concentrator plus, Eppendorf). The resulting dried residues were resuspended in 200 μL of methanol:water (4:1), vortexed, and centrifuged again at 15,000× *g* for 10 min at 4 °C, and again the supernatant fractions were collected and evaporated to dryness.

### 2.7. Mass Spectrometry Analysis of Plasma Metabolites

For Chromatography-Mass Spectrometry (GC-MS) analysis, a 100 μL aliquot of the metabolite sample was transferred to glass vials (1.5 mL) and lyophilized. Metabolite derivatization was performed according to previously described protocols [12]. In the following step, the sample was derivatized. For derivatization, 30 μL of methoxyamine (15 mg mL^−1^) in pyridine was added, agitated for 1 min, and then incubated for 16 h at room temperature in the dark. Silylation was performed by adding 30 μL of MSTFA (N-methyl-trimethylsilyl-trifluoroacetamide) with 1% TMCS (trimethylchlorosilane). Samples were left to stand for 1 h in the dark. Subsequently, 30 μL of Heptane was added. At this stage, a series of alkanes (C12–C40) was used, which allowed the calculation of retention time. Pooled QC samples, prepared by combining equal aliquots from all experimental samples, were periodically analyzed throughout the analytical run to monitor instrument performance and retention time stability.

The derivatized samples were automatically injected (1 μL) in splitless mode into a gas chromatograph 8890 (GC Agilent Technologie, Santa Clara, CA, USA), equipped with a DB-5 20 m long × 0.18 mm internal diameter × 0.18 μm film fused silica column (Agilent J&W Scientific, Folsom, CA, USA). The injection temperature was 280 °C, with a flow rate of 20 mL min^−1^, initiated after 300 s of data acquisition, with the initial temperature of the first column being 80 °C, held for 2 min and increased 15 °C/min until reaching 305 °C, then this temperature was held for 2 min. The column effluent was introduced into the ion source of the GC-TOFMS equipment (Pegasus BT, Leco, St. Joseph, MI, USA). The ion source temperature was 250 °C, electron beam 70-eV, ionization current of 2.0 mA and 20 spectra s^−1^ were recorded in the range of 45–800 *m*/*z*, and the detector voltage was 1500 V.

The GC-MS data were processed using the ChromaTOF for BT software, version 1.2.0.6, in which baseline correction, deconvolution, retention index (RI) acquisition, retention time (RT) correction, peak identification and alignment were performed, and metabolite identification was performed by using the NIST library, version 2.4 (year 2020). Valid identification required a mass spectral similarity score ≥800 against the NIST 2020 library and the presence of ≥3 characteristic *m*/*z* fragments.

### 2.8. Plasma Lipid Extraction

Plasma lipid extraction was performed using the Bligh and Dyer method [24]. Briefly, 50 µL of plasma was used as the starting material. Subsequently, 250 µL of methanol pre-cooled to −40 °C, 150 µL of water, and 250 µL of chloroform were added. The mixture was then centrifuged for 5 min at 16,000× *g* at 4 °C to promote phase separation. Following centrifugation, the lower organic phase containing the lipid fraction was carefully collected and dried under a gentle stream of nitrogen gas.

### 2.9. Plasma Lipidome Analysis

Dried lipid extracts were resuspended in 100 µL of chloroform:isopropanol (1:4, *v*/*v*). For positive ionization mode analysis, samples were further diluted 100-fold in the same solvent, whereas for negative mode analysis, samples were injected without dilution. Liquid chromatography–mass spectrometry (LC–MS) analysis was performed using a Nexera X2 UHPLC system (Shimadzu, Kyoto, Japan) equipped with a Zorbax Eclipse XDB-C18 column (50 × 4.6 mm, 1.8 µm particle size; Agilent) and coupled to a Maxis Impact ESI-QTOF mass spectrometer (Bruker Daltonics).

Chromatographic separation was performed using a binary gradient system consisting of mobile phase A (5 mM ammonium acetate, pH 5, in water for negative mode or 0.1% formic acid in water for positive mode) and mobile phase B (isopropanol). The gradient program was as follows: 0–1 min, 10% B; 1–10 min, linear increase to 80% B; 10–25 min, held at 80% B; 25–27 min, returned to 10% B; and 27–35 min, maintained at 10% B for column re-equilibration. The column temperature was maintained at 50 °C, with a constant flow rate of 0.3 mL/min.

Mass spectrometric detection was performed using the Maxis Impact ESI-QTOF (Bruker, Bremen, Germany) instrument equipped with an electrospray ionization source under the following conditions: nebulizer gas pressure of 5 bar, drying gas flow of 8 L/min, drying temperature of 220 °C, capillary voltage of 4500 V, and end plate offset of 800 V. Mass spectra were acquired in both positive and negative ion modes over an *m*/*z* range of 100–2000 at an acquisition rate of 1.33 Hz using a data-dependent acquisition (DDA) strategy. For each acquisition cycle, the five most intense precursor ions were selected for MS/MS fragmentation using stepped collision energies of 30 and 60 eV. Pooled quality control (QC) samples from each experimental group were also analyzed to support lipid identification based on MS/MS fragmentation patterns.

Data processing was performed using MZmine version 4.9 [25]. The workflow included noise detection, chromatogram building, chromatogram smoothing, feature resolving, 13C isotope filter, feature alignment and lipids.

### 2.10. Statistical Analysis

The Kolmogorov–Smirnov normality test assessed data distribution, with significant differences investigated using *t*-tests or chi-squared tests. The statistical analyses of the proteomic and metabolomics data were performed with the online MetaboAnalyst 6.0 program (http://www.metaboanalyst.ca/MetaboAnalyst/ (accessed on 10 April 2026)). For clinical data, statistical analyses were conducted within the R environment. To minimize missing values, molecular features, proteins, lipids, and metaboliteswere retained in the final matrix only if quantified in at least 50% of samples in at least one study group. To construct and evaluate integrative classification models, molecular features were selected based on statistically significant differential abundance following Benjamini–Hochberg False Discovery Rate correction (*q* < 0.05). Prior to multivariate modeling, abundance matrices for clinical parameters, proteomics, metabolomics, and lipidomics were normalized using Pareto scaling. The integrated multi-block modeling was performed using sparse Partial Least Squares Discriminant Analysis (block.splsda, mixOmics package version 6.26/MetaboAnalyst 6.0). Model hyperparameters, including the optimal number of components and selected variables per block, were tuned using repeated 10- foldcross-validation (repeated 10 times).

Quantitative analysis was performed using a one-way T test with MetaboAnalyst 6.0 to identify statistically significant differences (*p* < 0.05) in normalized abundance between groups. MetaboAnalyst 6.0 was also utilized for Partial Least Squares-Discriminant Analysis (PLS-DA) and Pattern Hunter (PH) analyses to identify group differences. Pearson’s correlation analysis performed through PH categorized associations as moderately correlated (0.5–0.7), highly correlated (0.7–0.9), or very highly correlated (0.9–1.0) [26]. The corrplot package (version 0.92) facilitates correlation analysis between proteins and clinical data [27].

Enrichment analyses of the proteomics, metabolomics, and lipidomics datasets were conducted using bioinformatics platforms [28,29,30]. Functional enrichment of differentially expressed proteins was performed using the Enrichr platform (https://maayanlab.cloud/Enrichr/ (accessed on 15 April 2026)). Metabolomic pathway enrichment and topology analyses were carried out using MetaboAnalyst 6.0. Lipidomic enrichment and lipid class analysis were performed using LipidSig 2.0 (https://lipidsig.bioinfomics.org).

To identify key features differentiating groups, two multivariate classification algorithms were employed: PLS-DA with VIP (variable importance in the projection) score and Random Forest [31]. Random Forest provides prediction of disease risk based on patient characteristics and identification of the most relevant features (proteins and metabolites in this case) for sample classification [32,33,34]. In PLS-DA, VIP scores ≥ 1 indicate important variables [35]. To run the performance model, the mixOmics package version 6.26 was used, resulting in Receiver Operating Characteristic curves (ROC), confusion matrices and error rates for protein and metabolite analyses, to confirm Random Forest results [36]. We evaluated the model’s performance via 10-fold cross-validation, repeated 10 times. In each interaction, we fit a block.splsda model using the pre-specified from our final object on cross-validated samples and then measure the prediction accuracy on the held-out samples. Significance Analysis of Microarrays (SAM) was used to assess the robustness of the proteomics and metabolomics data. Cross-validation was used to assess the universality of a predictive model. To avoid overfitting in high-dimensional matrices, PLS-DA models were validated using 10-fold cross-validation repeated 10 times, evaluating *Q*^2^ cumulative values for predictive significance. Random Forest classification performance was assessed via Out-of-Bag (OOB) error estimation. Group segregation was non-parametrically validated using PERMANOVA with 999 permutations.

## 3. Results

### 3.1. Demographic and Clinical Evaluation

The demographic and clinical characteristics of the participants are summarized in Supplementary Table S1. The Kolmogorov–Smirnov normality test confirmed that the variables followed a normal distribution. Overall, statistical differences were observed between the control group and the MetS group, such as age, blood pressure values, triglycerides, HDL, VLDL-cholesterol and LDL-cholesterol, besides parameters directly associated with the MetS diagnosis and pathology (BMI, waist and hip measurement, glycemia and HOMA-IR). Individuals in the MetS group exhibited a reduction in HDL-cholesterol levels along with an increase in the other parameters cited before.

**Figure 1:**
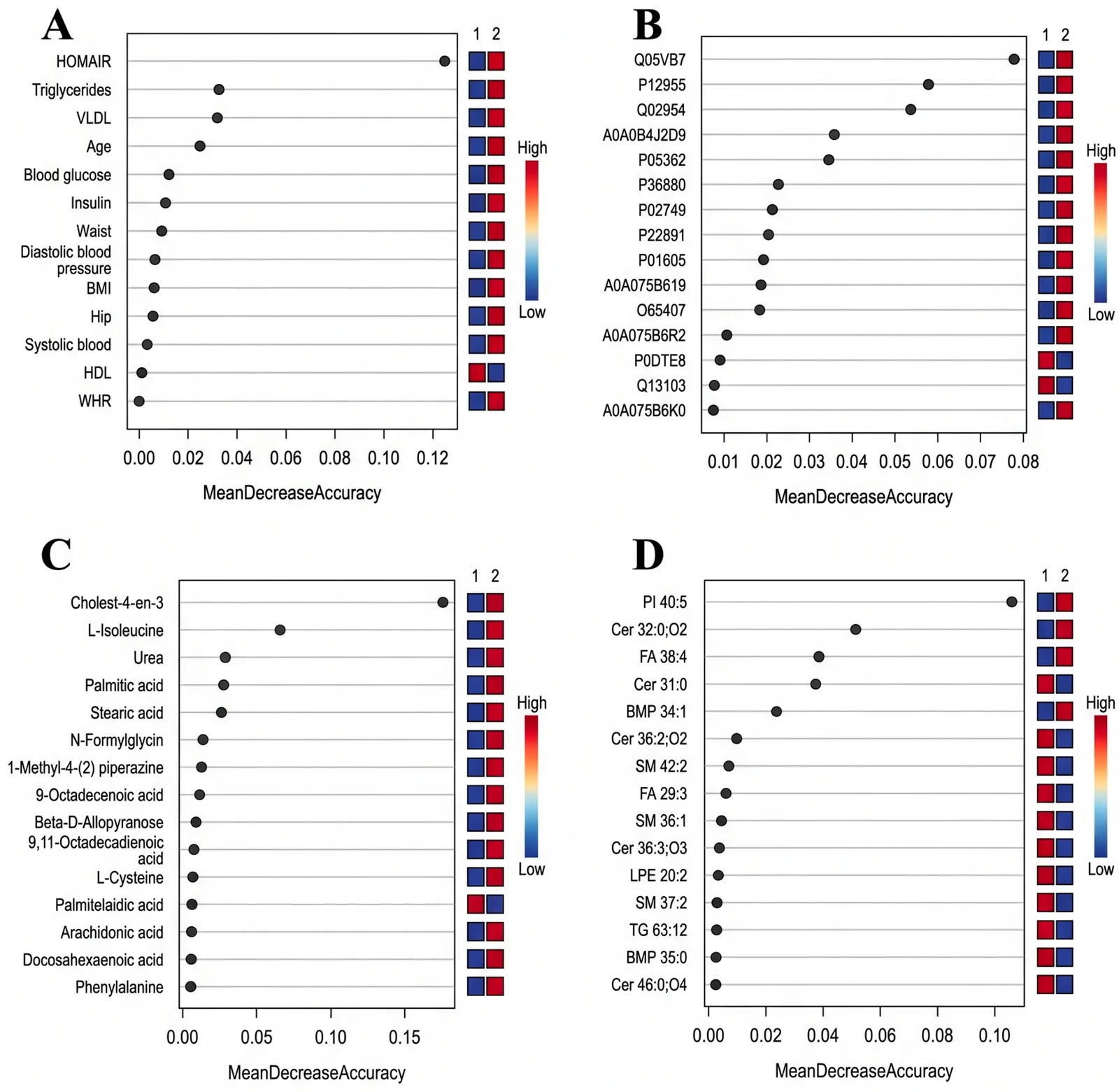
Feature importance ranked by Random Forest analysis. Plots display the Mean Decrease Accuracy for (**A**) clinical data, (**B**) proteomic features, (**C**) metabolomic features, and (**D**) lipidomic features. The mini-heatmap on the right illustrates the relative intensity/level of each feature.

Correlation analysis (Supplementary Table S2 and Supplementary Figure S1A) further supported the association between these variables and the case group. HOMA-IR showed the strongest positive correlation, followed by triglyceride levels and waist circumference. In contrast, HDL-cholesterol demonstrated a significant negative correlation, reinforcing its reduction as an important marker of the MetS-altered metabolic profile. To identify the clinical parameters that best discriminate between groups, a machine learning analysis using a Random Forest algorithm was performed (Supplementary Table S3 and Figure 1A). The Mean Decrease Accuracy results indicated that HOMA-IR was the most important predictive variable for group classification, followed by triglycerides, VLDL, and age.

### 3.2. Plasma Proteomic Profiles

An untargeted, gel-free quantitative mass spectrometry approach was employed to characterize proteomes. This strategy enabled the identification of 589 plasma proteins (post-filtering process), of which 26 showed significant differences in abundance between the studied groups (Supplementary Table S4). To characterize the proteomic profile that differentiates MetS from the control group, supervised partial least squares-discriminant analysis (PLS-DA) was employed. The score plot revealed a robust and distinct separation between the two cohorts along the primary components (Supplementary Figure S2A). The model demonstrated high predictive performance and reliability (R2 0.91972 and Q2 0.89295), as evidenced by cross-validation metrics reaching an accuracy of 0.967 for two components. This group segregation was statistically validated by PERMANOVA, which indicated a significant difference in protein profiles between the groups (F = 81.424, R2 = 0.584, *p* = 0.001). To identify potential outliers across the datasets, Principal Component Analysis (PCA) was performed on proteomic profiles (Supplementary Figure S3A) showing robust group separation along the first principal component, with only one outlier. Complementary machine learning analysis using Random Forest further corroborated these findings, yielding a low OOB error of 0.0333. Variable importance analysis, based on Mean Decrease Accuracy (MDA), identified a consensus panel of highly discriminatory proteins. Specifically, Scavenger receptor cysteine-rich type 1 protein M130, Xaa-Pro dipeptidase and Proteoglycan 4 emerged as the top contributors to the classification accuracy, as the Immunoglobulin kappa variable 1D-13 and Intercellular adhesion molecule 1 (Supplementary Table S5 and Figure 1).

Pearson correlation coefficients were utilized to evaluate the association between individual protein expression and the MetS phenotype. Several proteins exhibited strong positive correlations with the syndrome (Supplementary Figure S1B). Notably, Scavenger receptor cysteine-rich type 1 protein M130, Proteoglycan 4, and Intercellular adhesion molecule 1 showed the most significant associations, suggesting a coordinated upregulation of these features in the MetS cohort (Supplementary Table S6). To evaluate the predictive performance of the model based on proteomic signatures, a Receiver Operating Characteristic (ROC) curve analysis was performed. Even with only two variables, the model demonstrated robust performance, achieving an Area Under the Curve (AUC) of 0.908 (95% CI: 0.789–1.000). As model complexity increased to 26 variables, with the AUC increasing to 0.997 (95% CI: 0.974–1.000). These results indicate that the proteomic signature is highly effective in discriminating healthy individuals from patients with MetS, with only marginal error rates (Supplementary Figure S4A).

Finally, to resolve the biological pathways represented by the differentially expressed proteins, we performed a Reactome pathway enrichment analysis. The results indicated that the discriminatory proteomic signature is heavily centered on innate and adaptive immunity. The “Complement Cascade” and “Regulation of Complement Cascade” were the most significantly enriched terms, involving key proteins such as CRP, C7, CFHR2, and C4BPA (Supplementary Figure S5 and Supplementary Table S7).

### 3.3. Plasma Metabolomic Profiles

A total of 410 metabolites were identified in plasma samples, of which 27 exhibited differential abundance between the study groups (Supplementary Table S8). PLS-DA analysis revealed a robust separation between the two groups (Supplementary Figure S2B), exhibiting high predictive power and explanatory capacity (R2 0.94582 and Q2 0.91951), with cross-validation metrics yielding an accuracy 0.98571 for the two-component model. The statistical significance of this separation was further validated by PERMANOVA (F-value = 40.058; R2 = 0.408; *p* = 0.001). Random Forest analysis yielded an OOB error of 0.0167. The top Random Forest variable importance measures, where Cholest-4-en-3-ol and L-Isoleucine display the highest MDA scores of 0.178 and 0.063, respectively (Figure 1C and Table S9). Metabolomic performance was assessed by ROC curves, which indicated high sensitivity and specificity for the identified metabolic signatures (Supplementary Figure S4B). Potential multivariate outliers were evaluated using PCA. For metabolomic data (Supplementary Figure S3B), distinct group clustering was achieved; however, a single sample from the MetS group positioned itself as a marginal outlier at the far right of the *x*-axis.

Correlation analysis established distinct metabolic patterns associated with the MetS phenotype. Stearic acid and palmitic acid showed the strongest positive correlations with the clinical condition. Significant associations were also observed for some amino acids, specifically L-tyrosine, L-tryptophan and phenylalanine (Supplementary Table S10 and Supplementary Figure S1D). In contrast, 3-hydroxybutyric acid showed a negative correlation pattern. Quantitative Enrichment Analysis mapped these metabolic alterations to several pathophysiological pathways. The most significant enrichment was observed for Late-onset preeclampsia and Obesity. Other highly enriched pathways included 2-ketoglutarate dehydrogenase complex deficiency, colorectal cancer, and sepsis (Supplementary Table S11 and Supplementary Figure S6).

### 3.4. Plasma Lipidomic Profiles

A total of 1241 lipids were identified in plasma samples, of which 41 showed differential abundance between the study groups (Supplementary Table S12). PLS-DA revealed a robust separation between the two groups along the first two principal components (Supplementary Figure S2C). The predictive performance of the model was validated through cross-validation, yielding high accuracy (0.947, R2 0.89821 and Q2 0.82011). The statistical significance of this separation was further confirmed by PERMANOVA (F-value: 30.404; R-squared: 0.34392; *p*-value: 0.001), indicating distinct lipidomic signatures between the control and MetS groups. PCA was applied to the data to screen for multivariate outliers. The lipidomic profile (Supplementary Figure S3C) showed the highest within-group variance, reflecting a highly heterogeneous molecular distribution. Nonetheless, all samples clustered largely within their 95% confidence intervals, indicating that this dispersion represents intrinsic biological diversity rather than technical anomalies.

Random Forest classification was employed to identify the most discriminative lipid species, achieving an overall OOB error rate of 0.05. Analysis of the MDA identified PI 40:5 as the most influential feature for group discrimination, followed by Cer 32:0; O2 and FA 36:4 (Supplementary Table S13 and Figure 1D). Correlation pattern analysis further delineated the relationship between specific lipid species and the metabolic phenotype. PI 40:5 exhibited the strongest positive correlation with the MetS group, followed by Cer 32:0; O2 and BMP 34:1 (Supplementary Table S14 and Supplementary Figure S1D). Lipidomics performance was evaluated by ROC curves, demonstrating high sensitivity and specificity (Supplementary Figure S4C).

Lipidomic profiling of the MetS group revealed a profound remodeling of the lipid composition of plasma compared to the control group. Functional assessment indicated a significant raise of Phosphatidylcholine and Phosphatidylinositol classes in the case group. Conversely, a marked and highly significant depletion was observed in Sphingomyelin (Supplementary Figure S7A). Subcellular distribution analysis further characterized the possible localization across cellular components. The MetS group was characterized by a significant increase in lipidic pathways associated with the mitochondria, while there was a significant decrease in lipid related to multiple other organelles. Specifically, significant reductions were observed in the endosome/lysosome and Golgi apparatus (Supplementary Figure S7B).

### 3.5. Multi-Omics Evaluation

**Figure 2:**
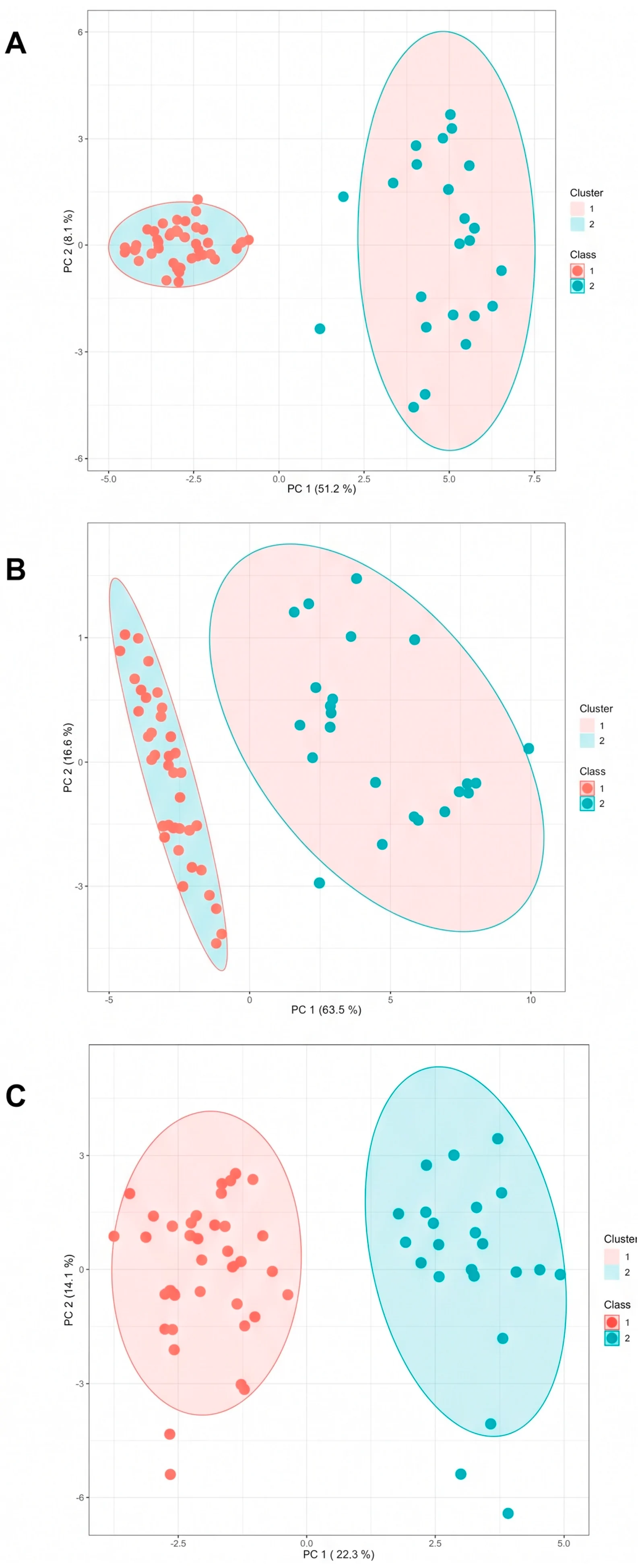
Unsupervised K-means clustering. (**A**) PCA score plot based on protein profiles. (**B**) PCA score plot of metabolite profiles. (**C**) PCA score plot of lipid profiles.

To verify the capacity of plasma molecular signatures to discriminate biological profiles and validate the experimental design, an unsupervised K-means clustering algorithm (k = 2) was applied across the proteomics, metabolomics, and lipidomics profiles. The results revealed a robust and clear separation between healthy controls and the metabolic dysfunction group considering all omics data, with metabolomics displaying the highest spatial segregation power (PC1 of 63.5), followed by proteomics (PC1 of 51.2) and lipidomics (PC1 of 22.3%). Crucially, all MetS volunteers were grouped cohesively and T2DM-related MetS and non-T2DM MetS individuals presented similar dispersion patterns. This spatial overlap demonstrates that both subgroups share a common and highly dysregulated molecular background (Figure 2).

To delineate the molecular landscape associated with MetS, we integrated proteomic, metabolomic, and lipidomic datasets within a comprehensive multi-omics framework. PLS-DA of the combined datasets revealed a clear separation between the Control and MetS cohorts, demonstrating high classification performance (Supplementary Figure S8). Hierarchical clustering analysis further delineated the differences between groups, revealing distinct molecular clusters associated with MetS status (Figure 1D). Additionally, the multi-omics heatmap highlighted specific proteins co-clustered with metabolic and lipid signatures, suggesting a coordinated systemic response in individuals with MetS.

**Figure 3:**
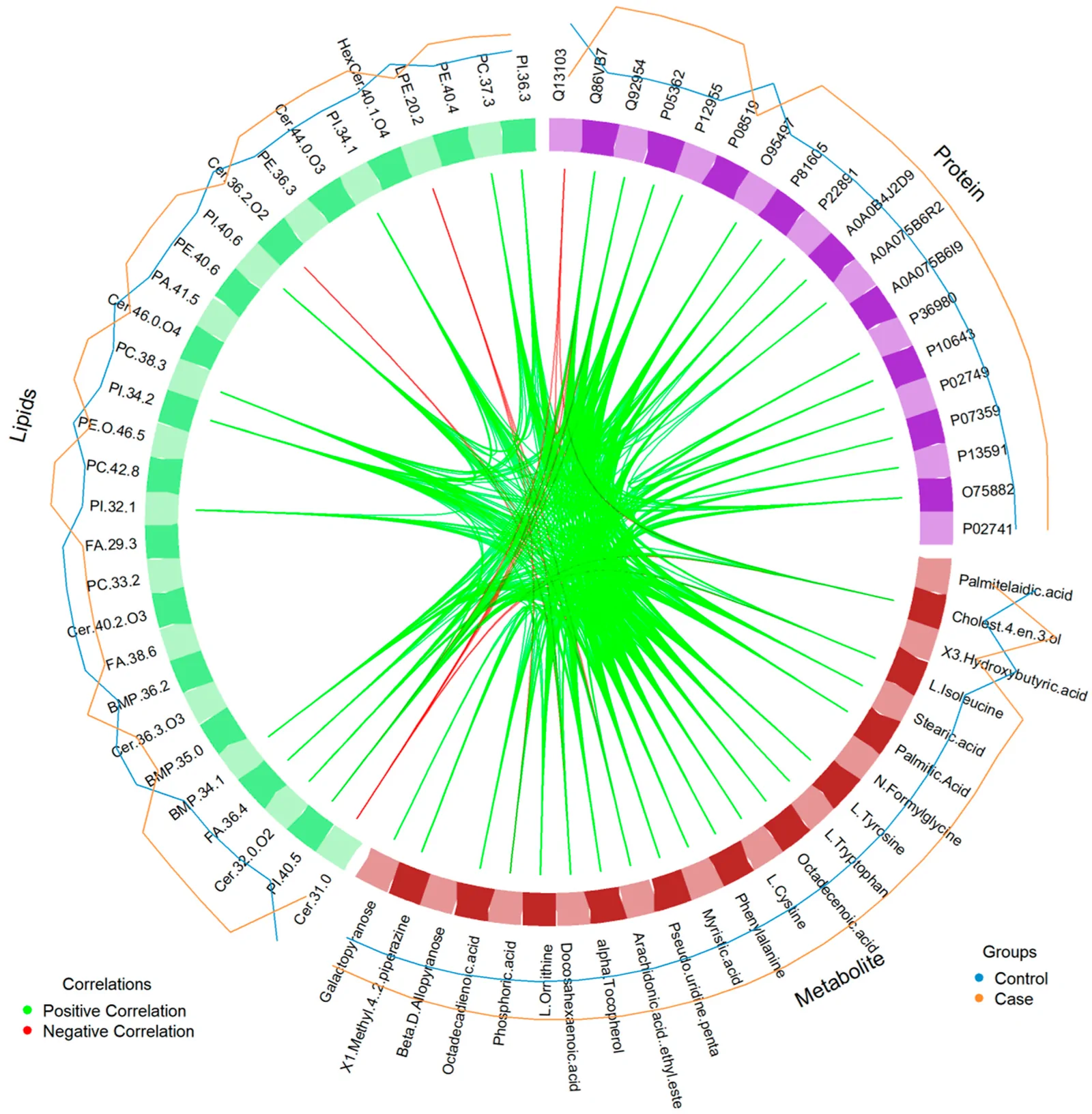
Multi-omics correlation Circos plot integrating proteins, lipids, and metabolites. The diagram displays the interconnected landscape of the identified molecular features. The perimeter is divided into three omics sectors (Proteins, Lipids, and Metabolites), with individual features labeled along the circumference. The outer line plots reflect the relative abundance trends in the Control (blue) and Case (orange) groups.

Integrative network analysis combining proteomics, metabolomics, and lipidomics data also revealed a highly interconnected molecular portrait distinguishing MetS from healthy profiles. The integrated network consists of 107 nodes, including 26 proteins, 27 metabolites, and 41 lipids, as well as 13 clinical phenotypic markers (Supplementary Table S15, Figure 3 and Supplementary Figure S9).

A central “metabolic syndrome cluster” was identified, characterized by strong positive correlations with BMI, HOMA-IR, and triglycerides. Within this cluster, the phosphatidylinositol species PI(40:5) emerged as a primary lipid hub, demonstrating a robust association with insulin resistance. This lipid node was significantly connected to several metabolic markers, particularly amino acids, with the strongest inter-omics correlations observed between PI(40:5) and L-cystine, phenylalanine, and L-tryptophan. Saturated fatty acids, including stearic acid and palmitic acid, showed high positive correlations with BMI, further defining this dysmetabolic signature.

Among the identified proteins, intercellular adhesion molecule 1 (ICAM-1, P05362) and scavenger receptor cysteine-rich type 1 protein M130 (CD163, Q86VB7) emerged as central inflammatory hubs. ICAM-1 exhibited strong positive associations with both BMI and HOMA-IR, and was highly correlated with several amino acids, such as L-cystine and phenylalanine. Similarly, CD163 showed significant connectivity with insulin resistance and triglycerides, suggesting a synchronized activation of inflammatory and metabolic pathways.

To evaluate the contribution of proteins, metabolites, and lipids to diagnostic performance, we constructed a series of predictive models using the MetaboAnalyst platform. We first established a baseline model (Model 1) comprising five conventional clinical variables (HOMA-IR, blood glucose, triglycerides, VLDL, and age). While Model 1 provided foundational classification performance, it exhibited significant overlap in predicted class probabilities (Supplementary Figure S10A). The corresponding confusion matrix highlighted major limitations, showing a substantial number of false negatives (n = 10) and false positives (n = 9), underscoring that traditional clinical markers alone are insufficient for high-resolution patient stratification.

Integrating proteomic features (Supplementary Figure S10B–E) yielded incremental improvements in model performance. This synergistic effect was most pronounced in Model 5 (comprising intercellular adhesion molecule 1, vanin-1, and apolipoprotein D), which significantly reduced misclassification compared with the clinical-only baseline model. Similarly, the addition of metabolites (Supplementary Figures S10F,G and S11A,B) and lipid species (Supplementary Figure S11C–F), specifically phosphatidylinositols (PIs), phosphatidylcholines (PCs), and sphingomyelins (SMs), markedly refined model specificity. Model 13 (Supplementary Figure S10F) emerged as a high-performing classifier, achieving a substantial reduction in total classification errors.

The most robust performance was achieved by the Integrated Global Model (Supplementary Figure S11G), which combined clinical data with selected protein, metabolite, and lipid signatures (intercellular adhesion molecule 1, vanin-1, apolipoprotein D, palmitic acid, 3-hydroxybutyric acid, L-isoleucine, L-tryptophan, L-tyrosine, phenylalanine, PI(32:1), PI(34:1), PI(34:2), PI(36:3), PI(40:4), PI(40:5), PI(40:6), PC(33:2), PC(33:3), PC(37:3), PC(38:3), PC(42:8), SM(30:1), SM(36:1), SM(37:2), and SM(42:2)). This model demonstrated near-perfect class separation in predicted class probability plots, with samples sharply segregated.

## 4. Discussion

Apolipoprotein D (ApoD), a glycoprotein and prominent member of the lipocalin family, functions as a critical multi-ligand transporter and a key mediator of systemic lipid homeostasis and antioxidant defense [37]. Unlike other apolipoproteins, ApoD is primarily associated with high-density lipoproteins (HDL) and exerts potent anti-inflammatory effects by modulating the oxidation of arachidonic acid and sequestering small hydrophobic molecules that trigger oxidative stress [38]. Within the context of metabolic disorders, ApoD has been identified as a protective factor, whose expression is typically induced under cellular stress to mitigate lipid peroxidation and neutralize pro-inflammatory signaling [38]. The reduction in ApoD levels observed in our MetS group suggests a systemic failure of this protective mechanism against the oxidative insult inherent to the syndrome, thereby facilitating the transition from simple insulin resistance to overt vascular dysfunction [39,40]. Consequently, the loss of this anti-inflammatory shield correlates with the disrupted lipid profiles observed clinically, suggesting that restoring or maintaining ApoD levels could be a viable therapeutic strategy for the MetS phenotype [38,39,40].

Vanin-1 (VNN1) plays a pivotal role at the interface of lipid metabolism and inflammatory responses [40]. Its primary biological function involves the hydrolysis of pantetheine into pantothenic acid (vitamin B5), a process that directly influences cellular oxidative stress balance [12,41]. The clinical relevance of VNN1 in the pathophysiology of insulin resistance and T2DM is evidenced by its capacity to modulate pro-inflammatory signaling pathways, such as the iNOS/MCP-1/TGF-beta axis, exacerbating systemic and tissue inflammation in individuals with obesity [42]. Furthermore, elevated VNN1 levels have been associated with higher glycated hemoglobin (HbA1c) levels and the progression of cardiovascular complications, suggesting that its upregulation is a critical event in the transition from simple obesity to established MetS [41,43]. Consequently, understanding the mechanisms by which VNN1 integrates metabolic and inflammatory signals is essential for developing targeted therapeutic strategies.

Intercellular adhesion molecule 1 (ICAM-1) is a transmembrane glycoprotein belonging to the immunoglobulin superfamily that plays a pivotal role in modulating immune responses and maintaining vascular integrity [44]. It is predominantly expressed on endothelial cells and leukocytes, where it facilitates the leukocyte adhesion cascade that culminates in cellular transmigration into inflamed tissues. However, in response to pro-inflammatory stimuli, its upregulation serves as a hallmark event of endothelial dysfunction [45]. The detection of ICAM-1 exclusively in our MetS cohort, in contrast to its absence in healthy controls, reflects a fundamental shift in vascular homeostasis driven by an adverse metabolic environment. Evidence demonstrates that individuals with the metabolically unhealthy obesity phenotype exhibit significantly elevated levels of this protein compared with healthy individuals, a finding that directly correlates with increased visceral adiposity and reduced levels of protective adipokines, such as adiponectin, which normally inhibits adhesion molecule expression [45,46]. Therefore, these findings position ICAM-1 as a central pathophysiological mediator linking metabolic dysregulation to the progression of systemic vascular damage [44,46].

The metabolic landscape of MetS is increasingly defined by systemic shifts in branched-chain amino acids (BCAAs) and aromatic amino acids (AAAs) [47,48]. In this study, we observed significantly elevated levels of L-isoleucine, L-tyrosine, phenylalanine, and L-tryptophan in the MetS cohort compared with healthy controls. Specifically, L-isoleucine accumulation correlates with increased BMI and adiposity, whereas its restriction is known to restore insulin sensitivity through the secretion of fibroblast growth factor 21 (FGF21) [47]. Impaired BCAA catabolism hiperactivates the mTORC1 pathway, leading to the inhibitory phosphorylation of IRS-1 and subsequent insulin resistance [49,50]. Simultaneously, elevated AAAs such as phenylalanine and L-tyrosine exacerbate metabolic decline by promoting lipid disorders through increased hepatic bile acid synthesis and by contributing to visceral fat accumulation and oxidative stress [51,52]. Furthermore, L-tryptophan dysregulation, driven by chronic low-grade inflammation, accelerates progression toward type 2 diabetes [53,54]. Together, these findings underscore a synergistic role for BCAAs and AAAs, positioning these molecular profiles as valuable tools for early risk stratification and as potential therapeutic targets [55].

Palmitic acid (PA) is the most prevalent saturated fatty acid in human circulation, representing approximately 20% to 30% of total fatty acids in membrane phospholipids and adipose tissue triacylglycerols [56,57]. Our findings reveal a significant upregulation of circulating PA in patients with MetS compared with healthy controls. This elevation reflects a systemic state of lipid overload strongly associated with chronic low-grade inflammation and insulin resistance [58]. Furthermore, our observation of PA enrichment aligns with recent evidence demonstrating that PA promotes pro-inflammatory M1 macrophage polarization and adipose tissue dysfunction [59]. Beyond its role in glucose dysregulation, elevated PA levels in MetS likely contribute to heightened cardiovascular risk by increasing atherosclerotic plaque vulnerability and the risk of premature coronary artery disease [60]. The obesogenic potential of PA is further underscored by its ability to accelerate lipid accumulation and inflammatory responses during early adipogenesis, exacerbating the expansion of dysfunctional white adipose tissue [61]. Taken together, the marked elevation of PA in individuals with MetS may represent a potent lipotoxic driver bridging obesity, systemic inflammation, and T2DM [57].

3-Hydroxybutyrate (3HB) is a low-molecular-weight ketone body synthesized endogenously in the liver that acts as a pleiotropic signaling molecule central to the regulation of metabolic and inflammatory homeostasis [62]. Our results reveal a significant reduction in serum 3HB levels in individuals with MetS compared with healthy controls, suggesting a loss of its protective effects against insulin resistance and the aggravation of glucose intolerance in T2DM [62]. Furthermore, the 3HB deficiency observed in the MetS group may exacerbate chronic low-grade inflammation and oxidative stress, as physiological concentrations of this metabolite exert anti-inflammatory properties that mitigate the activation of systemic inflammatory pathways in models of metabolic dysfunction [62]. The clinical relevance of this finding is reinforced by evidence showing that elevated fasting 3HB levels are positively associated with weight loss success in intervention programs, serving as a marker of metabolic health and metabolic flexibility—the body’s capacity to efficiently switch between fuel sources [63]. In summary, the reduction in 3HB identified in our study points to the impairment of essential protective signaling pathways, positioning the restoration of 3HB levels as a promising therapeutic strategy, as well as a valuable tool for risk stratification and monitoring the progression of systemic metabolic disorders [8,62].

Phosphatidylinositol (PI) is a pivotal anionic phospholipid that serves as both a fundamental structural component of cellular membranes and a precursor for essential phosphoinositide signaling molecules, such as PIP3, which orchestrates the PI3K/Akt pathway central to insulin action and glucose homeostasis [64]. In the present study, a distinct molecular signature characterized by the significant upregulation of specific PI species was identified in individuals with MetS compared with healthy controls. This accumulation of PIs suggests a remodeling of the systemic lipidome that aligns with increased adiposity and metabolic dysfunction. Crucially, recent evidence from a comprehensive Mendelian randomization analysis has established a causal link between genetically predicted PI levels and a 17% increased risk of MetS, providing strong support for the hypothesis that the PI elevations observed in our study are drivers of the disease state rather than merely secondary consequences [12]. These findings are intricately tied to the pathophysiology of insulin resistance and chronic low-grade inflammation, where dysregulated PI concentrations may exacerbate glucose intolerance and progression toward T2DM [65,66,67]. Collectively, these results position these PI species as critical metabolic alterations. Their systemic elevation underscores the metabolic dysregulation underlying MetS pathogenesis, highlighting PI metabolism as a promising therapeutic target for restoring insulin sensitivity and mitigating systemic metabolic risk.

Phosphatidylcholine (PC) is the most abundant phospholipid in eukaryotic membranes and a primary constituent of circulating lipoproteins. In our study, a significant upregulation of the PC class was observed in individuals with MetS compared with healthy controls. This elevation in circulating PC levels is strongly associated with the development of insulin resistance, T2DM, chronic low-grade inflammation, and mitochondrial stress, often serving as an early indicator of metabolic deterioration [68,69]. The expansion of the PC pool in the context of MetS has been linked to impaired insulin signaling. Specifically, recent evidence suggests that high-fat diet–induced increases in exosomal PC can inhibit the insulin receptor pathway in hepatocytes and macrophages via the activation of the aryl hydrocarbon receptor (AhR), thereby driving systemic inflammation and glucose intolerance [70]. In a study by Waś et al. [71], PC shifts in individuals at high cardiovascular risk served as sensitive indicators of preclinical cardiovascular changes, suggesting that the PC imbalances found in our MetS cohort may directly herald an increased susceptibility to major adverse cardiovascular events. Clinically, the elevation of PC levels in the MetS group underscores the profound lipid dysregulation characterizing this syndrome and highlights the therapeutic importance of targeting phospholipid remodeling pathways to mitigate progression toward overt diabetes and cardiovascular disease.

Sphingomyelins (SMs) are primary phosphosphingolipids in mammalian cells, serving as integral structural components of plasma membranes and key regulators of cholesterol homeostasis and protein trafficking [72,73]. Our study revealed a significant decrease in circulating SM species in individuals with MetS compared with healthy controls. Our findings align with specific lipidomic signatures reported in pediatric populations, where decreased levels of specific SM species were strongly associated with abdominal obesity and glucose dysregulation, linking this depletion to the pathogenesis of insulin resistance and T2DM [72,74,75]. Furthermore, SM administration in mouse models of obesity has been shown to reduce inflammatory cytokine levels, significantly lower total hepatic lipids, triglycerides, and total cholesterol, and improve the overall glycemic profile [75]. Targeting this specific lipidomic alteration or restoring SM levels may provide a promising avenue for risk stratification and the prevention of cardiovascular complications in individuals with MetS [75,76].

By adopting a systems-level multi-omics framework, this study transcends traditional single-analyte paradigms to demonstrate that MetS represents a synchronized molecular breakdown across distinct biological layers rather than a mere aggregate of independent risk factors. Our integrated network reveals a profound homeostatic collapse where proteomic, lipidomic, and metabolomic alterations directly intersect to drive systemic insulin resistance, tissue lipotoxicity, and vascular vulnerability. A central mechanistic connection emerging from our data is the direct cross-talk between circulating lipid excess and endothelial inflammatory activation. Elevated levels of circulating palmitic acid (PA) [57,58,59], a saturated fatty acid, serve as an endogenous ligand for Toll-like receptor 4 (TLR4), triggering downstream IKK/NF-κB transcriptional cascades [57,59]. This signaling axis directly induces the endothelial expression and surface upregulation of ICAM-1 [45,46,47], initiating the leukocyte adhesion and transmigration cascade characteristic of early endothelial dysfunction [45,46]. This pro-inflammatory state is further amplified by the upregulation of vanin-1 (VNN1) [40,41,42,43,44], an enzyme that drives pantetheine hydrolysis to increase tissue oxidative stress and exacerbate the pro-inflammatory iNOS/MCP-1/TGF-beta signaling axis [43,77]. Simultaneously, this lipotoxic-inflammatory state directly intersects with dysregulated amino acid catabolism and disrupted membrane phosphoinositide signaling. Systemic accumulation of branched-chain amino acids (BCAAs, particularly L-isoleucine) [48,50,51] and aromatic amino acids (AAAs) [52,53,54,55,56] induces the mechanistic target of rapamycin complex 1 (mTORC1) pathway [11,50]. Chronic mTORC1 hyperactivation promotes inhibitory serine phosphorylation of insulin receptor substrate 1 (IRS-1) [50,51], thereby uncoupling the canonical insulin receptor/PI3K/Akt signaling axis [50,65]. Crucially, this signaling blockade directly converges upon the observed remodeling of circulating membrane phospholipids—specifically, the marked elevation of phosphatidylinositol (PI) [65,66,67,68,69] and phosphatidylcholine (PC) [70,71,72,73] species. As phosphoinositides function as vital secondary messengers in PI3K/Akt signaling [12,65], their dysregulated accumulation reflects impaired membrane signaling dynamics under severe insulin resistance [12,67], while elevated PC species further suppress hepatic insulin receptor activation [72]. This metabolic stress is exacerbated by the concomitant loss of circulating sphingomyelins (SMs) [74,75,76], which normally exert protective, anti-inflammatory, and lipotropic functions [75,77]. This multi-faceted pathogenic cycle is severely compounded by the collapse of endogenous antioxidant and anti-inflammatory shields. Physiologically, apolipoprotein D (ApoD) [37,38,39,40] acts as a lipocalin transporter that neutralizes lipid peroxidation and restrains systemic inflammation, while 3-hydroxybutyrate (3HB) [63,64] mitigates metabolic stress through PPAR-alpha activation [63]. The significant depletion of both ApoD [37,38,39,40] and 3HB [63,64] deprives metabolic and vascular tissues of crucial physiological buffers, allowing PA-induced TLR4 activation and BCAA-mediated mTORC1 stress to proceed unchecked. While these findings must be interpreted within the constraints of a cross-sectional design, which captures multi-omics profiles at a single time point and precludes establishing direct causality, the extensive multivariate cross-validation and strong inter-omics correlations underscore a synchronized molecular collapse defining MetS pathophysiology. Although the moderate cohort size warrants caution regarding generalizability, this multi-layered synthesis provides a comprehensive map linking nutrient excess, endothelial injury, and membrane remodeling to guide future targeted therapeutic interventions.

## 5. Conclusions

This study reinforces the concept that MetS represents a synchronized molecular alteration across proteomic, lipidomic, and metabolomic axes, rather than a simple collection of independent risk factors. The findings highlight a profound homeostatic breakdown characterized by the depletion of protective compounds, such as apolipoprotein D (ApoD) and sphingomyelin species, which compromises cellular antioxidant and anti-inflammatory defenses. This vulnerability is further exacerbated by the elevation of pro-inflammatory mediators such as ICAM-1 and vanin-1, the accumulation of lipotoxic drivers such as palmitic acid, and a significant disruption in branched-chain amino acid (BCAA) catabolism that stimulates the mTORC1 pathway. Coupled with the loss of the metabolic signaling molecule 3-hydroxybutyrate and the upregulation of specific phosphatidylinositol and phosphatidylcholine species, these shifts collectively drive systemic insulin resistance and vascular damage. While acknowledging the limitation of a moderate cohort size, this study leveraged high-dimensional, multi-layered omics profiling supported by extensive multivariate statistical validations and cross-validation pipelines. Rather than aiming to propose or validate definitive clinical biomarkers, our primary objective was to unravel the deeper, integrated pathophysiology of metabolic syndrome. Ultimately, this multi-omics landscape provides a comprehensive framework to understand the systemic complexity of MetS and guide future translational research.

## Data Availability

The raw mass spectrometry data are available from the corresponding author upon reasonable request and with the author’s permission.

## Supplementary Materials

The following supporting information can be downloaded at: https://www.mdpi.com/article/10.3390/biology15201838/s1, Figure S1: Feature correlation analysis across clinical, protein, metabolite, and lipid profiles.; Figure S2: Partial Least Squares-Discriminant Analysis (PLS-DA) score plots.; Figure S3: Principal Component Analysis (PCA) score plots of plasma proteins, metabolites and lipids; Figure S4: ROC curves for predictive evaluation of plasma proteins, metabolites and lipids; Figure S5: Overview of enriched metabolite sets; Figure S6: Pathway enrichment analysis of identified proteins; Figure S7: Abundance levels across lipid classes and sub-cellular component distributions; Figure S8: Multi-omics integration score plots for sample separation.; Figure S9: Hierarchical clustering heatmap of multi-omics molecular signatures; Figure S10: Predicted class probability distributions and cross-validation confusion matrices (Model Evaluation Set 1).; Figure S11: Predicted class probability distributions and cross-validation performance (Model Evaluation Set 2); Table S1: Demographic and clinical data of the 60 volunteers (mean ± sd); Table S2: Correlation table of clinical data; Table S3: Random Forest mean decrease accuracy of clinical data; Table S4: Differentially abundant proteins identified between the case and control cohorts; Table S5: Random Forest mean decrease accuracy of plasma protein; Table S6: Correlation table of plasma protein; Table S7: Reactome pathway enrichment analysis; Table S8: Differentially abundant metabolites identified between the case and control cohorts; Table S9: Random Forest mean decrease accuracy of plasma metabolites; Table S10: Correlation table of plasma protein; Table S11: Enrichment analysis of plasma metabolites; Table S12: Differentially abundant lipids identified between the case and control cohorts; Table S13: Random Forest mean decrease accuracy of plasma lipids; Table S14: Correlation table of plasma lipids; Table S15: Correlation matrix integrating multi-omics profiles and clinical parameters.
