# Supplementary material for: Integrative Multi-Omics Reveal the Molecular Architecture and Systemic Burden of Metabolic Syndrome in a Brazilian Cohort
Source: Biology (Basel). 2026 Oct 14;15(20):1838. doi: 10.3390/biology15201838 (PMC13701559; doi:10.3390/biology15201838)
Supplement: Supplementary file 1 [file biology-15-01838-s001.zip › Supplementary Figure S8.pdf]

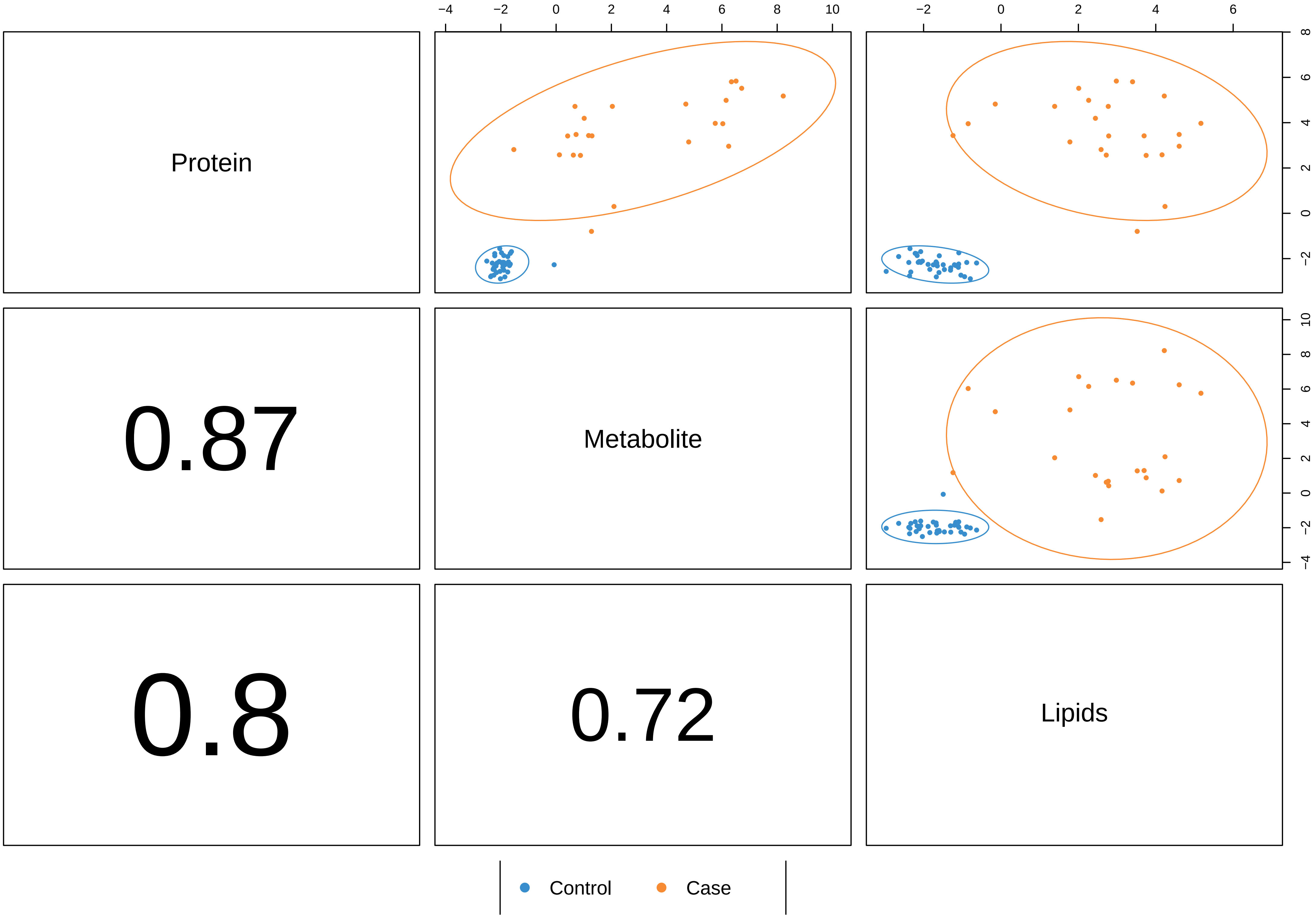

Protein
Metabolite
Lipids
0.87
0.8
0.72
Control
Case
