# Supplementary material for: Integrative Multi-Omics Reveal the Molecular Architecture and Systemic Burden of Metabolic Syndrome in a Brazilian Cohort
Source: Biology (Basel). 2026 Oct 14;15(20):1838. doi: 10.3390/biology15201838 (PMC13701559; doi:10.3390/biology15201838)
Supplement: Supplementary file 1 [file biology-15-01838-s001.zip › Supplementary Figure S9.pdf]

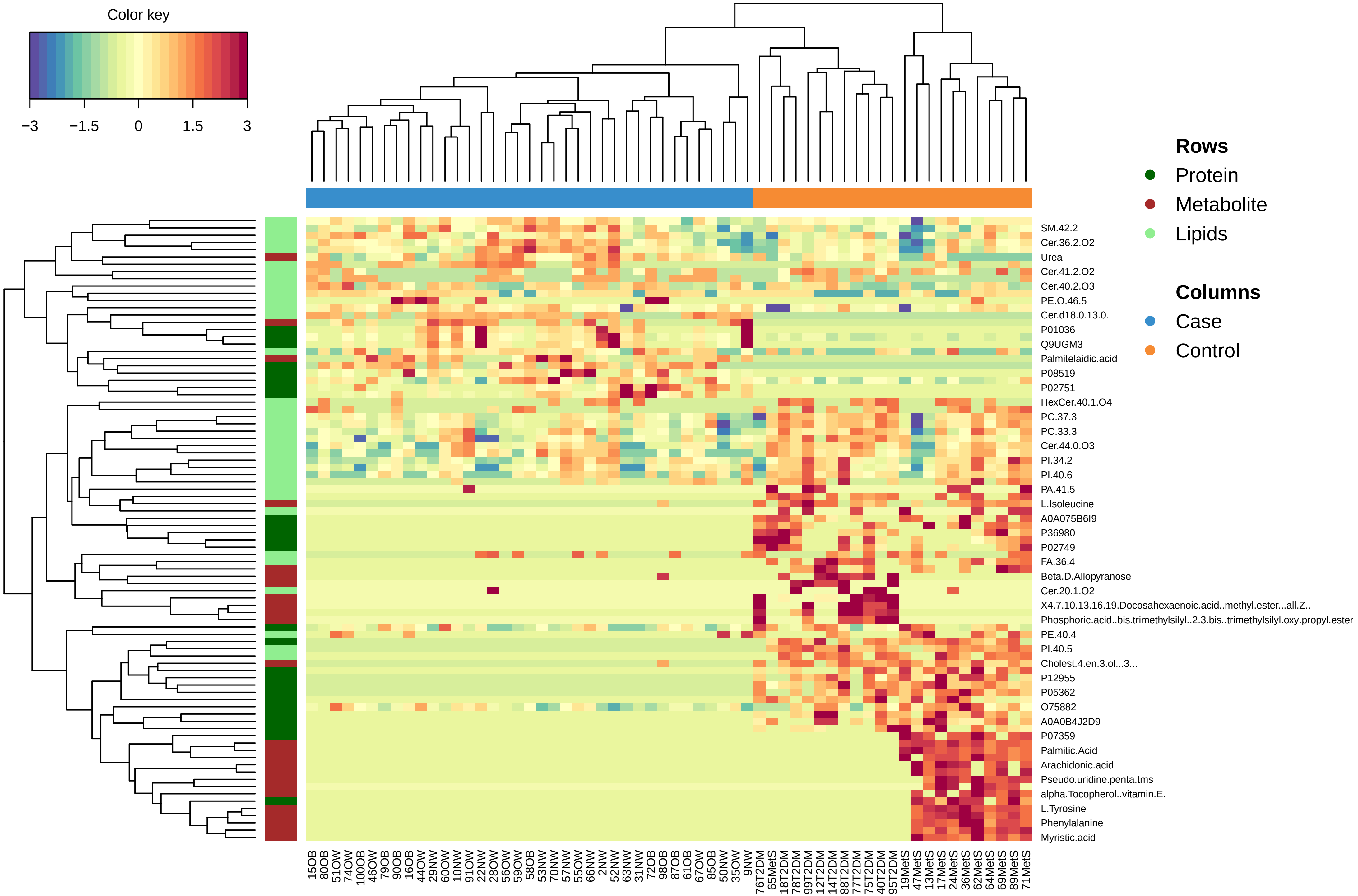
Color key
−3
−1.5
0
1.5
3
Rows
Protein
Metabolite
Lipids
Columns
Case
Control
SM.42.2
Cer.36.2.O2
Urea
Cer.41.2.O2
Cer.40.2.O3
PE.O.46.5
Cer.d18.0.13.0.
P01036
Q9UGM3
Palmitelaidic.acid
P08519
P02751
HexCer.40.1.O4
PC.37.3
PC.33.3
Cer.44.0.O3
PI.34.2
PI.40.6
PA.41.5
L.Isoleucine
A0A075B6I9
P36980
P02749
FA.36.4
Beta.D.Allopyranose
Cer.20.1.O2
X4.7.10.13.16.19.Docosahexaenoic.acid..methyl.ester...all.Z..
Phosphoric.acid..bis.trimethylsilyl..2.3.bis..trimethylsilyl.oxy.propyl.ester
PE.40.4
PI.40.5
Cholest.4.en.3.ol...3...
P12955
P05362
O75882
A0A0B4J2D9
P07359
Palmitic.Acid
Arachidonic.acid
Pseudo.uridine.penta.tms
alpha.Tocopherol..vitamin.E.
L.Tyrosine
Phenylalanine
Myristic.acid
15OB
80OB
51OW
74OW
100OB
46OW
79OB
90OB
16OB
44OW
29NW
60OW
10NW
91OW
22NW
28OW
56OW
59OW
58OB
53NW
70NW
57NW
55OW
66NW
2NW
52NW
63NW
31NW
72OB
98OB
87OB
61OB
67OW
85OB
50NW
35OW
9NW
76T2DM
65MetS
18T2DM
78T2DM
99T2DM
12T2DM
14T2DM
88T2DM
77T2DM
75T2DM
40T2DM
95T2DM
19MetS
47MetS
13MetS
17MetS
24MetS
36MetS
62MetS
64MetS
69MetS
89MetS
71MetS
